# Oromandibular Dystonia and Dysphagia in Hyperglycemic Brainstem Dysfunction

**DOI:** 10.5334/tohm.1062

**Published:** 2025-08-05

**Authors:** Gero Lueg, Timm Westhoff, Martin Fruth, Regina Kerkmann, Julia Krämer

**Affiliations:** 1 Department of Geriatric Medicine, Marien Hospital Herne, Ruhr University Bochum, Herne, Germany; 2 Medical Department I, Marien Hospital Herne, Ruhr University Bochum, Bochum, Germany; 3 Department of Diagnostic, Interventional Radiology and Nuclear Medicine, Marien Hospital Herne, Ruhr University Bochum, Herne, Germany; 4 Department of Neurology with Institute of Translational Neurology, University Hospital Münster, Münster, Germany

**Keywords:** Brainstem dysfunction, Diabetic Striatopathy, Hyperglycemic Movement Disorder, Hyperglycemia, Dysphagia, Oromandibular Dystonia, Osmotic Demyelination

## Abstract

**Background:**

Hyperglycemia-induced movement disorders usually present as hemichorea or hemiballismus. Non-choreiform presentations are rare and often overlooked.

**Case Report:**

We present the case of a 36-year-old man with uncontrolled type 2 diabetes who developed painful oromandibular dystonia, dysarthria and dysphagia. These symptoms were investigated using flexible endoscopic evaluation of swallowing (FEES). An MRI revealed reversible T2 hyperintensities in the pons without striatal involvement. The symptoms resolved with insulin normalization and tetrabenazine treatment.

**Discussion:**

Transient brainstem dysfunction due to hyperglycemia may present with oromandibular dystonia and dysphagia. FEES facilitates early detection of subtle yet clinically relevant complications.

## Background

Prolonged severe hyperglycemia has been shown to result in a broad spectrum of movement disorders, most commonly hyperkinetic presentations such as chorea-hemiballismus syndrome. Despite an increasing number of case reports documenting non-choreiform neurological symptoms, these cases still account for less than a third of the reported manifestations of hyperglycemia-related movement disorders [1,2]. These hyperglycemia-related movement disorders are predominantly categorized under the broader umbrella terms of diabetic striatopathy and hyperglycemic hyperosmolar syndrome [3]. Two theories have been postulated to explain this complex neurological syndrome: (i) dysfunctional degradation of γ-aminobutyric acid (GABA), impairing projection neurons within the basal ganglia, and (ii) hyperglycemia-induced hyperosmolarity, causing reactive astrogliosis, as seen on magnetic resonance imaging (MRI) [4]. However, only approximately 55% of patients with hyperglycemia-associated movement disorders exhibit characteristic MRI findings, such as T1-weighted striatal hyperintensity, as microstructural network lesions remain undetectable in standard sequences [1,5]. This case presents a young patient with hyperglycemic induced acute hyperkinetic movement disorder characterized by jaw-closing dystonia and associated dysphagia. Both the clinical symptoms and the pontine lesions regressed over the course of the disease.

## Case report

A 36-year-old male patient with a medical history of type 2 diabetes mellitus presented to the hospital with a two-day history of painful facial muscle cramps as well as difficulties with eating and drinking. Laboratory tests revealed a blood glucose level of 484 mg/dl (26.9 mmol/L), calculated serum osmolality of 320 mosmol/kg, serum sodium of 130 mmol/L. HbA1c was 16.1%, C-peptide was normal, serum creatinine was documented as 1.6 mg/dl (141.4 µmol/L), accompanied by the presence of glucosuria and proteinuria and trace ketones in the urine, but no evidence of acidosis with a blood pH of 7.47.

**Figure 1:**
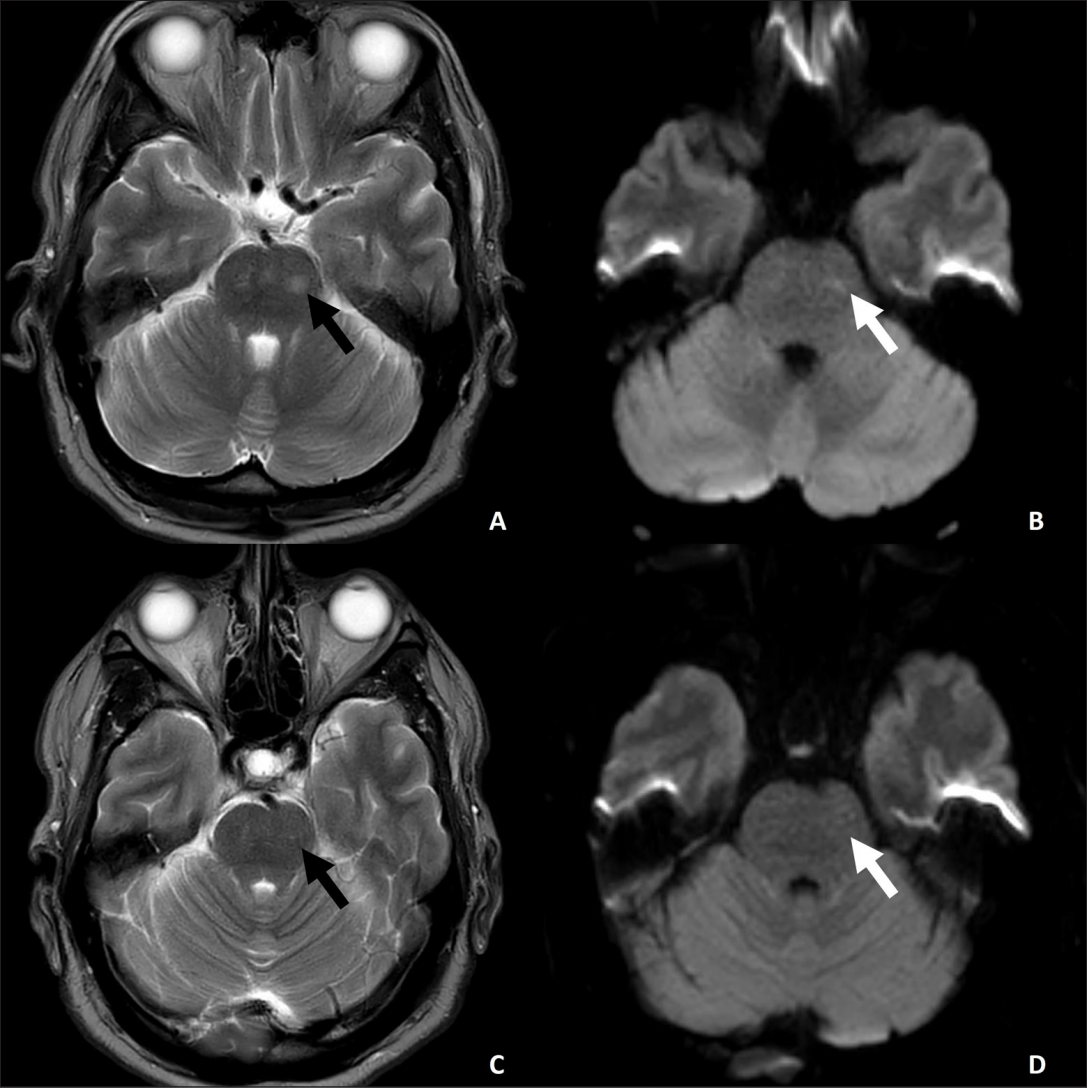
Magnetic resonance imaging of the pons. **(A)** Axial T2-weighted image shows bilateral, ill-defined hyperintensities in the central pons. **(B)** Diffusion-weighted imaging (b1000) demonstrates no evidence of restricted diffusion, and the corresponding ADC map (not shown) indicates preserved diffusion, suggesting a vasogenic component. **(C)** and **(D)** Follow-up imaging four weeks later shows complete resolution of the hyperintensities. The imaging pattern, in combination with clinical reversibility, is consistent with reversible pontine dysfunction due to osmotic injury. While the findings favor vasogenic edema, a mild form of osmotic demyelination syndrome (ODS) cannot be entirely excluded.

The initial MRI (see Figure 1) revealed the presence of bilateral hyperintense lesions (A) in the T2-weighted sequences, suggesting the occurrence of acute gliosis or edema. The axial diffusion-weighted image b1000 (B) demonstrated minimal vasogenic edema in the center of the largest lesion on the left side (arrows in A/B). No evidence of T1-weighted signal hyperintensities was observed in the striatal brain regions or the caudate nucleus and putamen (data not shown).

As demonstrated in Additional File 1, Segment 1, the patient did not present with the characteristic symptoms typically observed in hyperglycemia-related movement disorders, such as hemiballismus or hemichorea. However, the patient exhibited features that are suggestive of segmental oromandibular dystonia. The patient exhibited symptoms consistent with jaw-closing dystonia, a specific form of oromandibular dystonia. In this case, the manifestation of the condition resulted in involuntary contraction of the masseter muscle, which was accompanied by the involvement of the tongue and mimic muscles. This led to recurrent tongue and cheek biting and difficulties with eating and drinking. The patient reported task-specific exacerbation of symptoms, including dysarthria and dysphagia, during activities such as drinking or speaking.

In Segment 2 of Additional File 1, the patient is shown during fiberoptic endoscopic examination of swallowing (FEES). During the attempt to drink water, the patient exhibited involuntary, brief, painful jaw closure. During the water swallowing test, the patient is only able to swallow the bolus in small amounts and in an uncontrolled manner due to the dystonic involvement of the tongue and the jaw muscles. This results in a prolonged oral phase, impaired bolus control, and multiple swallowing attempts. These deficits result in pre-deglutitive aspiration, which is cleared by a forceful protective cough. While no pharyngeal dystonia was observed, the aspiration pattern and preserved cough response suggest transient sensory impairment of the pharynx, likely due to pontine dysfunction.

Segment 3 presents the patient seven days after receiving adequate insulin therapy and taking 75 milligrams of tetrabenazine daily. A complete regression of jaw-closing dystonia and hyperkinetic movements is already evident at this point. Segment 4 presents the patient on May 14, 2025, four weeks after the initial admission to the hospital. Notably, no neurological abnormalities were observed, even after the discontinuation of tetrabenazine two weeks prior. The follow-up MRI scan (see Figure 1) revealed the complete resolution of the vasogenic edema (D) and the nearly complete resolution of the pontine T2-weighted hyperintense lesions (C). These results confirm the potential reversibility of the aforementioned lesions, which is consistent with the observed clinical course.

## Discussion

This case illustrates a young patient with an atypical clinical presentation of a secondary, hyperglycemia-associated movement disorder featuring jaw-opening and -closing dystonia accompanied by oropharyngeal dysphagia confirmed by FEES—a rare method of documentation in this context. The neurological symptoms were linked to a transient pontine lesion indicative of vasogenic edema. The clinical and radiological findings appeared only in the context of a hyperosmolar hyperglycemic state and disappeared completely after metabolic stabilization.

The pontine localization of the lesion is notable because symptoms of the brainstem are rarely reported in hyperglycemia-related neurological syndromes [1]. However, the pons is vulnerable to osmotic stress because of its dense, inflexible arrangement of oligodendrocytes, which can impair local osmotic compensation mechanisms [6]. Additionally, the anatomical interface between gray and white matter, coupled with the pontine region’s high vascularization, facilitates the entry of myelinotoxic substances or promotes vasogenic edema when the blood–brain barrier is disrupted [6,7]. These pathophysiological features may explain why osmotic injury, whether vasogenic or demyelinating, affects the central pons more than other brain regions.

The pontine localization of the lesion is notable because symptoms of the brainstem are rarely reported in hyperglycemia-related neurological syndromes. However, the pons is vulnerable to osmotic stress because of its dense, inflexible arrangement of oligodendrocytes, which can impair local osmotic compensation mechanisms. Additionally, the anatomical interface between gray and white matter, coupled with the pontine region’s high vascularization, facilitates the entry of myelinotoxic substances or promotes vasogenic edema when the blood–brain barrier is disrupted. These pathophysiological features may explain why osmotic injury, whether vasogenic or demyelinating, tends to affect the central pons more than other brain regions.

Several reports have described osmotic demyelination syndrome (ODS), including both central pontine and extrapontine myelinolysis, in the context of hyperosmolar hyperglycemia, even in the absence of hyponatremia or rapid sodium correction [7,8]. Although dystonia is more commonly reported in ODS secondary to hyponatremia, movement disorders with craniofacial involvement have been observed following osmotic shifts due to hyperglycemia [2,5,9]. Importantly, increasing evidence suggests that non-choreiform hyperkinetic symptoms, including dystonia, can occur in hyperosmolar states without radiological signs of demyelination [1,3,5]. This indicates transient neuronal dysfunction or microstructural lesions below the resolution of conventional MRI.

The bilateral T2-hyperintense pontine lesions observed in our patient raise the question of whether they are indicative of vasogenic edema or demyelination. Several radiological and clinical features support the interpretation of a transient, non-demyelinating process. First, the lesions appeared symmetrical and lacked sharply demarcated borders or the classical imaging signs, such as the “trident sign” or “piglet sign,” that are typically seen in central pontine myelinolysis [10]. Second, although not shown, diffusion-weighted imaging (DWI) revealed no diffusion restriction, and apparent diffusion coefficient (ADC) maps showed increased values, which are more consistent with vasogenic than cytotoxic edema [8,11]. Third, complete radiological and clinical resolution, including sustained symptom remission following discontinuation of tetrabenazine therapy, underscores the reversible nature of the observed lesion pattern [12].

Notably, osmotic pontine edema is usually observed as an early, potentially reversible stage of osmotic injury, often occurring during the initial phase of ODS, before irreversible myelinolysis develops [13]. These conditions share a common pathophysiological basis of osmotic stress, astrocytic dysfunction, and blood-brain barrier disruption, but differ in severity and outcome [6]. In our case, the absence of enhancement, restricted diffusion, or neurological sequelae supports the interpretation of transient vasogenic edema rather than established ODS. This distinction is clinically relevant because early identification of reversible pontine osmotic injury can prevent progression to irreversible myelinolysis by allowing for the timely correction of metabolic derangements [6,8,11]. A further notable feature of this case is the presence of oropharyngeal dysphagia, which was documented using FEES. To our knowledge, this is the first report employing FEES in a hyperglycemia-associated movement disorder. The dysphagia was primarily related to oromandibular dystonia affecting the oral phase and triggering of the swallowing reflex. Although no dystonic pharyngeal movements were observed, recurrent microaspirations suggest a coexisting transient sensory deficit due to pontine dysfunction. Dysphagia has been reported in hyperosmolar states and ODS, usually resulting from bulbar involvement [14]. However, no previous reports have applied FEES to these conditions. Thus, this case may represent the first documented FEES-based assessment of dysphagia in a hyperglycemia-associated movement disorder.

## Conclusion

This case illustrates a rare manifestation of a hyperglycemia-associated movement disorder involving transient brainstem dysfunction, confirmed by FEES and reversible pontine lesions on MRI. Full recovery is possible with early metabolic correction, especially if the lesion reflects reversible osmotic injury rather than structural demyelination.

## Data Accessibility Statement

Additional data are available from the corresponding author on reasonable request.

## Additional File

The additional file for this article can be found as follows:

10.5334/tohm.1062.s1 Additional File 1. Segment 1 shows a patient with hyperkinetic movement disorder with involuntary contraction of the masseter muscle (jaw-closing dystonia) which led to recurrent tongue and cheek biting and dysarthria. Segment 2 demonstrates notable fractionation of water swallowing with consecutive pre-deglutitive aspiration in the fiberoptic endoscopic swallowing examination (FEES) due to tongue dystonia. In Segments 3 and 4, clinical remission is observed within one to four weeks after adequate insulin therapy and treatment with tetrabenazine.

## Abbreviations

FEES – Fiberoptic Endoscopic Evaluation of Swallowing, GABA – Gamma-Aminobutyric acid, MRI – Magnetic resonance imaging, T1w – T1-weighted MRI sequence, T2w – T2-weighted MRI sequence
